# New UK Chapter of the Alliance for a Cavity-Free Future

**DOI:** 10.1038/s41415-021-3730-4

**Published:** 2021-12-17

**Authors:** Nigel B. Pitts, Avijit Banerjee

**Affiliations:** Faculty of Dentistry, Oral and Craniofacial Sciences, King´s College London, Tower Wing, Guy´s Hospital, London, SE1 9RT, UK

## Abstract

The Alliance for a Cavity-Free Future (ACFF), started in 2010, has been based at King's College London since 2013. It is a dental caries-focused charity promoting integrated clinical and public health action. ACFF Chapters seek to improve caries prevention and management locally, based on best global evidence. The UK Chapter has been created in response to a continuing need combined with opportunities including the implementation of the latest UK version of the *Delivering better oral health* guidance on prevention (version 4). The Chapter has been formed through a coordinating committee with UKwide representation combining expertise in dental caries management across the patient life course. This committee co-created the Chapter Values Statement: 'in pursuit of a cavity-free future across the UK we value: collaboration and innovation; realistic and implementable prevention-based solutions; approaches that reduce health inequalities; action across both oral and general health settings; and working comprehensively from the population through to the individual level.' The agreed Chapter focus is on advocating for the implementation of appropriate, effective, pragmatic caries prevention and care across three themes: 1) in everyday dental practice; 2) in health and social care curricula; and 3) for vulnerable people across their life course on the basis of increased caries risk/susceptibility.

## Key points

- The Alliance for a Cavity-Free Future is a dental caries-focused advocacy charity seeking to confront and reduce the disease burden of caries through prevention.
- The UK Chapter, the latest of 29 'local' ACFF Chapters active in over 50 countries, seeks to improve caries prevention and management based on best global evidence.
- The initial focus is on actions: 1) in everyday dental practice; 2) in health and social care curricula; and 3) for vulnerable people across the life course.

## Introduction

The Alliance for a Cavity-Free Future (ACFF) started in 2010 and has been based at King's College London since 2013. It is a dental caries-focused public health and clinical advocacy charity based in the UK, seeking to promote integrated clinical and public health action to confront and reduce the disease burden of caries and cavities through prevention (www.acffglobal.org). With this new Chapter, ACFF now has 29 local Chapters active in over 50 countries, all seeking to improve caries prevention and management in locally-appropriate ways which are based on shared best global evidence. ACFF has had foundational support from Colgate since its inception, which is provided on a Corporate Social Responsibility basis without restrictions. A key activity occurring for over more than a decade has been to secure more timely implementation of research findings into both policy and practice, in order to improve health, care and outcomes at the individual and country levels.^1^ The ACFF works with the International Caries Detection and Assessment System (ICDAS) Foundation and other international partners as part of a wider Global Collaboratory for Caries Management umbrella group.^1^ Further details of how this involves resources such as the International Caries Classification and Management System (ICCMS) and the CariesCare International 4D System for use in practice are described elsewhere.^2^

## Why a UK Chapter of Alliance for a Cavity-Free Future now

Compared to many countries, the UK has been well provided for, with many organisations advocating caries prevention and the ACFF has had no desire to duplicate what was already being addressed. However, the new UK Chapter has been created in response to an unusual opportunity where a continuing need to tackle caries and cavities is evident at the same time as post-pandemic plans for oral and dental services are being designed and put in place in the UK, while at the international level, efforts for prevention in caries and oral health are aligning.^3^ A further timely opportunity has been the combination of the development of a new, UKwide, fourth version of the *Delivering better oral health* (*DBOH* v4) guidance on prevention,^4^ combined with an appetite from the former Public Health England to seek proactive implementation of this guidance. The new Chapter seeks to build on much that has been achieved in the caries space over the last decades through a range of international collaborations.^1^^,^^2^

## Who is involved and what has been the process so far?

**Fig. 1:**
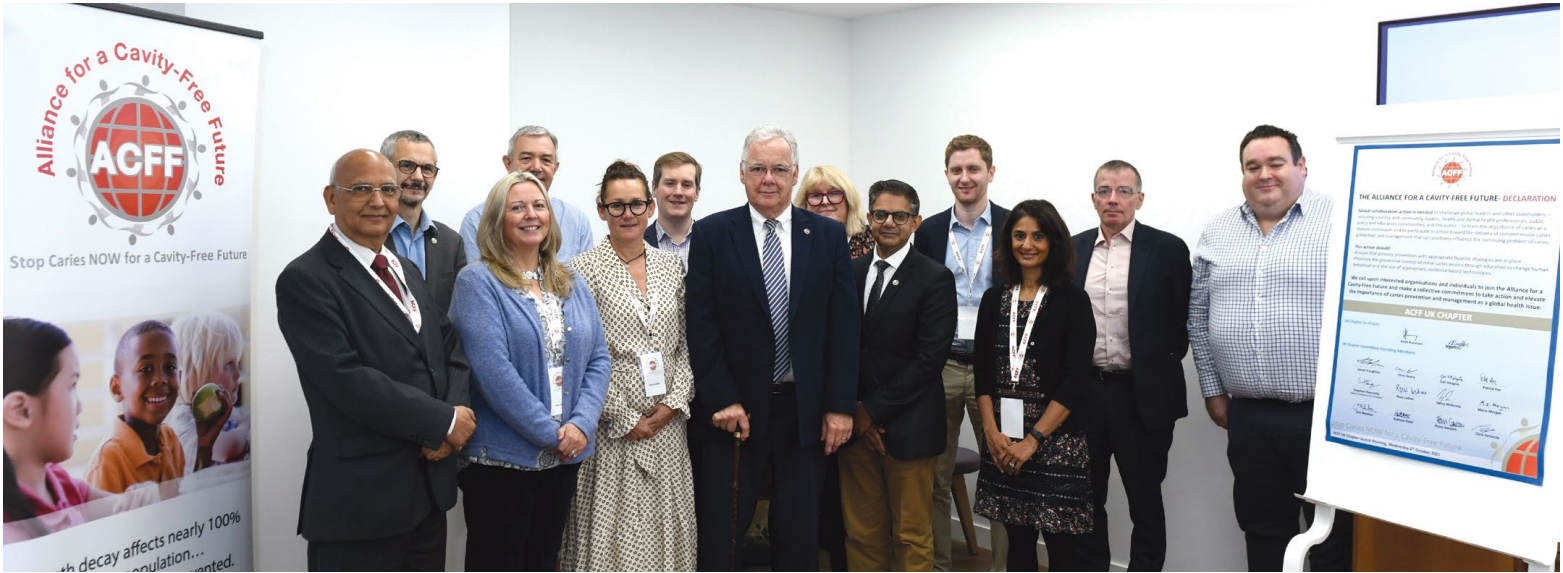
The Coordinating Committee meeting in London and signing the ACFF Declaration. The 2021 ACFF UK Chapter Coordinating Committee left to right - Russ Ladwa, Tim Newton, Gail Douglas, Stephen Hancocks, Fiona Sandom, Christopher Vernazza, Nigel Pitts, Maria Morgan, Avijit Banerjee, Patrick Fee, Rakhee Patel, Chris Deery and Gerry McKenna

Table 1 lists the 2021 ACFF UK Chapter coordinating committee members who were invited to join by the two co-chairs. They are shown (with one exception) in Figure 1 at a launch meeting held in London on 6 October 2021 alongside the ACFF UK Chapter Declaration they all signed at the meeting (Box 1). These Declarations have proved to be both popular and effective for many other ACFF Chapters. The coordinating committee members combine expertise in dental caries management across patients' life courses, as well as UKwide representation and membership of broad UK and international networks.

**Table 1:** The 2021 ACFF UK Chapter Coordinating Committee

Name	Institution
Avit Banerjee	King's College London (co-chair)
Nigel Pitts^*^	King's College London (co-chair)
Chris Deery	University of Sheffield; Dental Schools Council
Gail Douglas	University of Leeds
Patrick Fee	University of Dundee
Russ Ladwa	Immediate past president, British Dental Association
Gerry McKenna	Queen's University Belfast
Maria Morgan	Formerly Cardiff University
Tim Newton^*^	King's College London
Rakhee Patel	Public Health England; King's College London
Fiona Sandom	Health Education and Improvement Wales
Christopher Vernazza^*^	Newcastle University
Stephen Hancocks	British Dental Journal (communications consultant to Committee)
James Coughlan	Immediate past president, European Dental Students Assocation
Key: ^*^ = Alliance for a Cavity-Free Future Charity trustees

Box 1 ACFF UK Chapter declaration 'Global collaborative action is needed to challenge global leaders and other stakeholders - including country and community leaders, health and dental health professionals, public policy and education communities and the public - to learn the importance of caries as a disease continuum and to participate in action toward the delivery of comprehensive caries prevention and management that can positively influence the continuing problem of caries.

This action should:

- Ensure that primary prevention with appropriate fluoride strategies are in place
- Promote the preventive control of initial caries lesions through education to change human behaviour and the use of appropriate, evidence-based technologies.

We call upon interested organisations and individuals to join the Alliance for a Cavity-Free Future and make a collective commitment to take action and elevate the importance of caries prevention and management as a global health issue.'

## Co-created values

**Fig. 2:**
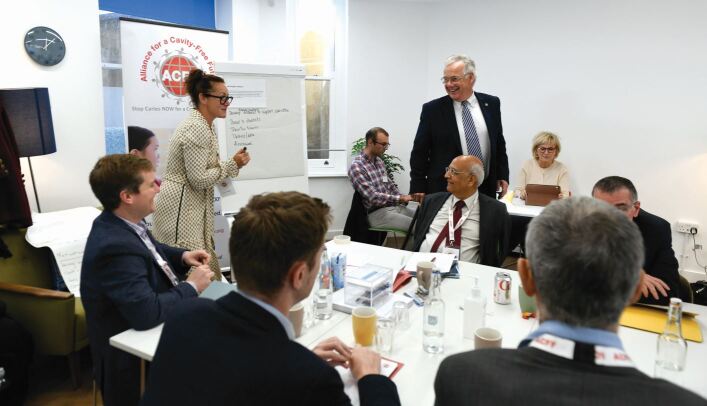
Coordinating Committee members in work groups co-creating values and focus/goals/themes

**Fig. 3:**
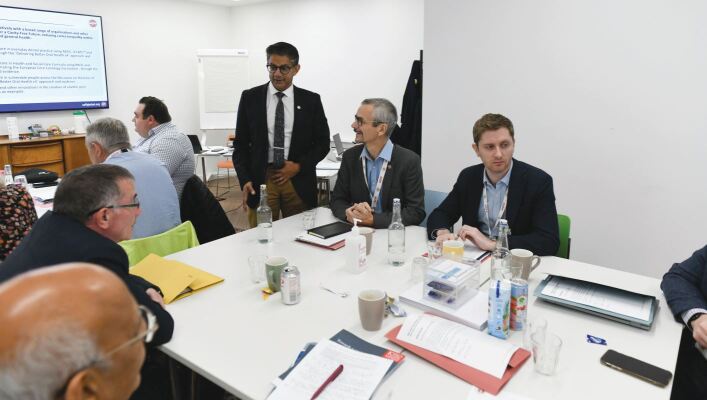
Coordinating Committee members in work groups co-creating values and focus/goals/themes

This committee has agreed both a Values Statement to guide its plans and actions as a Chapter and has then outlined three themes which should ideally all be taken forward. These tasks were accomplished after the distribution of background materials and an online discussion through facilitated face-to-face discussions and group work over the course of a one-day meeting. The sub-groups interacting in the discussions with support from the Chapter co-chairs is shown in Figures 2 and 3. The co-created and agreed ACFF UK Chapter Values Statement is:

In pursuit of a cavity-free future across the UK we value:

- Collaboration and innovation
- Realistic and implementable prevention-based solutions
- Approaches that reduce health inequalities
- Action across both oral and general health settings
- Working comprehensively from the population through to the individual level.

## What will the Chapter do?

### Determination of priority areas

Building and developing from an initial plan supplied by the co-chairs, the Committee after debate came to an agreed Chapter focus on 'advocating for the implementation of appropriate, effective, pragmatic caries prevention and care across three themes':

1. In everyday dental practice
2. In health and social care curricula
3. For vulnerable people across the life course on the basis of increased caries risk.

Before setting out the detail of the themes, it is important to understand how these can be underpinned by the hard work carried out by the former Public Health England (PHE) led team, who have reviewed the international evidence on prevention in oral health to produce the just published *DBOH* v4.

### *Delivering better oral health* version 4

The first version of *DBOH,* published in 2007, was produced as a collaboration between the then Department of Health and the British Association for the Study of Community Dentistry in order to support teams to provide evidence-based prevention. It sought to apply research evidence to give practical advice and has been a 'living' document, updated regularly with revisions in 2009, (led by PHE) 2014 and 2017.

*DBOH* v4 was published on 21 September 2021 by PHE (which now evolves into the new Office for Health Improvement and Disparities [OHID]). The fourth edition represents the work of a UKwide collaboration of well-respected experts and frontline practitioners, including patient representatives. It follows best practice in guideline development and the methodology has been published for transparency.

Evidence has been reviewed by systematic review experts Cochrane Oral Health and NICE-accredited clinical guidance development specialists, the Scottish Dental Clinical Effectiveness Programme. Dental caries was one of the Five Guideline Development Groups with topic experts from across the UK who came together to consider the evidence on specific topics and agree any changes from previous versions. The ACFF UK Chapter has agreed with PHE and its successor organisation OHID, to specifically help in the practical implementation of the caries sections of *DBOH* v4, while also supporting the wider oral health aspects where possible. The Chapter is represented on the formal *DBOH* v4 Implementation Group, alongside representatives of all four UK health departments.

### What's new in *Delivering better oral health* version 4?

*DBOH* v4 has been developed through a wider UK collaboration of experts, frontline practitioners and for the first time, patient representatives and is intended for use throughout dentistry in the UK. *DBOH* v4 has been published on the UK government website in a new digital format, making it easily accessible on mobile devices (although other digital forms may also be useful to some stakeholders in the future).

The summary tables have not changed significantly but have some new content - prevention of dental caries, for example, has new content on infant feeding. The importance of effectively supporting behaviour change is acknowledged and there is a greater consideration of older people and other vulnerable groups. Following an extensive review of the evidence, it is reassuring to note that there were no major changes to the advice offered or the professional interventions recommended to prevent oral diseases. A greater emphasis in this version has been placed on risk-based management including monitoring through appropriate dental recall and across the life course.

### The key overview of caries and supporting references used in *Delivering better oral health *version 4

Dental caries is one of the most prevalent non-communicable diseases (NCDs) nationally^5^^,^^6^ and globally.^7^ The disease is caused by dietary sugars that are broken down by microorganisms in the biofilm on a tooth surface which produces acids that, over time, demineralise tooth enamel.^8^^,^^9^

The process of demineralisation and remineralisation is dynamic.^8^ In the early stages of the disease, dental caries can be reversed. However, when factors promoting demineralisation exceed those favouring remineralisation, dental caries progresses (unless checked) into dentine, to a point where the tooth surface breaks down and ultimately a cavity forms.^9^

Effective patient care involves first diagnosing the presence and recording the extent of disease, using contemporary dental caries management tools such as the ICCMS,^10^ encouraging a reduction of factors that cause demineralisation, notably sugar consumption and enhancement of those favouring remineralisation, particularly the availability of fluoride and mineral ions. This may be achieved by a combination of preventive actions taken by patients, patient carers and healthcare professionals, supported by higher-level actions that promote policies and active change to facilitate a less cariogenic social environment.

### Early detection and management pathways

Given that dental caries can be identified and is reversible at an early stage, lesions should be identified at an early stage and managed. There is no evidence that a specific dental recall interval influences development or progression of dental caries.

The time between dental recall consultations, or 'check-ups', should be based on risk/susceptibility as assessed by the clinician working with patients (and where appropriate parents or guardians) and will be influenced by preventive care needed. The recall period will change across the patient's life course.

### The tools to be used in implementation by the UK Chapter

The following section on the three themes will make reference to the tools to be utilised by the UK ACFF Chapter for each theme. It is worth pointing out that it has been agreed by the Committee that the systems and implementation strategies developed in minimum intervention oral care (MIOC) delivery framework^11^ and the implementation lessons learned through ICCMS^10^ and the CariesCare International 4D consensus^12^^,^^13^ (described in detail elsewhere) will be employed across all three themes. The Chapter also wishes to reach out to other organisations, stakeholders and networks with shared aims as a true collaborative alliance.

It should also be appreciated that caries is now recognised as a NCD^14^ and links between the reduction in risk factors for the prevention of caries/cavities and other NCDs are clear.^14^^,^^15^ This means that caries prevention can also bring benefits to the prevention of obesity, diabetes and cardiovascular disease, as well as to other areas of oral health. This fits very well with the recent World Health Assembly Resolution on Oral Health.^16^

## Chapter focus in three idealised themes - the agenda

Boxes 1, 2 and 3 set out the agreed detail for how the UK ACFF Chapter hopes to make a positive impact on caries and cavities across the priorities it has identified. This is the start of the journey for the Chapter; there is much to do and we are as yet unsure about the resources and assistance that will be available to us. However, we feel it is helpful to share our professional aspirations and seek to achieve the stated aims by working in collaboration with OHID and a range of other professional organisations and networks as a true alliance - working with other organisations, not in competition with them.

### Theme 1 - advocating for the implementation of appropriate, effective, pragmatic caries prevention and care in everyday dental practice

The Chapteragreed that the priority is to focus on the reality of 'everyday' dental practice and to design implementation toolkits, education resources and motivation aids to bring caries prevention and management to this key audience. In doing so, the Chapter is fully aware, through the work of the three King's College London/ACFF Dental Policy Labs,^17^^,^^18^^,^^19^ of the policy-linked, economic and behavioural challenges of making this happen. Further challenges include accessible updated resources on modern minimum intervention oral care and minimally invasive operative cariology^2^ and assessing the behaviour and activity of initial lesions in enamel.^20^ The aim and ideal deliverables for this theme are set out in Box 2.

Box 2 Theme 1 - aim, ideal deliverables and resources to be employed Advocating for the implementation of appropriate, effective, pragmatic caries prevention and care in everyday dental practice.

Aim: to enhance the capability and motivation of healthcare practitioners to deliver prevention and create opportunities for appropriate, effective, pragmatic caries prevention and care through working in line with our values to enhance knowledge and skills, devising and disseminating strategies to create a culture of prevention and ensuring that systems recognise and reward prevention.

Ideal deliverables:

- Toolkits
- Education
- Motivation.

The delivery of this aim will utilise:

- MIOC, ICCMS and the CariesCare International 4D strategies
- The '*Delivering better oral health *v4' approach and evidence
- Other emerging strategies.

### Theme 2 - advocating for the implementation of appropriate, effective, pragmatic caries prevention and care in health and social care curricula

For this theme, the Chapter felt strongly that, although a key focus is the undergraduate dental curriculum and ensuring that the framework built over the last decade in Europe^21^^,^^22^^,^^23^ is available and shared across UK dental schools, it is important to also push the educational net much wider to dental and other healthcare teams and to key others in social care. The aim and ideal deliverables for this theme are set out in Box 3.

Box 3 Theme 2 - aim, ideal deliverables and resources to be employed Advocating for the implementation of appropriate, effective, pragmatic caries prevention and care in health and social care curricula.

Aim: to develop flexible resources to support the implementation of appropriate, effective, pragmatic caries prevention and care in health and social care curricula.

Ideal deliverables:

- Resources for healthcare students
- Resources for training educators and trainers
- Methods for appropriate assessment of skills acquired.

The delivery of this aim will utilise:

- MIOC, ICCMS and the CariesCare International 4D strategies
- The '*Delivering better oral health* v4' approach and evidence
- The European Core Cariology Curriculum
- Other emerging strategies.

### Theme 3 - advocating for the implementation of appropriate, effective, pragmatic caries prevention and care in vulnerable people across their life course on the basis of increased caries risk/susceptibility

A unifying view for this theme was that, as a first step, we need to raise awareness of the preventability of caries in specific and targeted ways with key stakeholder groups. A common approach to understanding how increased caries risk/susceptibility affects diverse vulnerable groups across different age ranges and settings was felt to be required. This would then be built on with mapping, engagement and co-production of implementable resources. There was also a strong desire to reach out beyond traditional dental networks to progress this theme. The aim and ideal deliverables for this theme are set out in Box 4.

Box 4 Theme 3 - aim, ideal deliverables and resources to be employed Advocating for the implementation of appropriate, effective, pragmatic caries prevention and care in vulnerable people across their life course on the basis of increased caries risk/susceptibility.

Aim: to raise awareness of the preventability of caries and draw out core guidance (from the evidence) for key stakeholder groups.

Ideal deliverables:

- Mapping of and engagement with key stakeholders in relation to prevention of caries in vulnerable groups
- Advocacy for the importance and impact of poor oral health on vulnerable groups
- Identification of core actions for preventive care for each vulnerable group
- Co-production of implementable resources specific to high-risk groups.

The delivery of this aim will utilise:

- MIOC, ICCMS and the CariesCare International 4D strategies
- The '*Delivering better oral health* v4' approach and evidence
- Other emerging strategies.

## Conclusion

The new UK Chapter of the ACFF has mapped out its values and ideal plan for taking forward three themes to advance a cavity-free future. The Chapter hopes to be able to provide 'how to' resources to help reduce the areas of disconnect between the current evidence and what is taught and what is actually done and paid for in the caries prevention and management space.

The Chapter is building a website which can be used to house a repository of useful material to share with all as a 'one stop shop'. As we plan the next steps, we would like to reach out to a range of wider networks, organisations and stakeholders (including appropriate industry partners working on a no restrictions basis) who can work with us on this challenging but important task.
